# *Azemiops feae* (Fea’s Viper) Envenoming: A Case Report and Review of the Literature

**DOI:** 10.3390/toxins18050201

**Published:** 2026-04-26

**Authors:** Zichen Qiao, Yong Tang, Qianshun Zhou, Bryan G. Fry

**Affiliations:** 1 Adaptive Biotoxicology Lab, School of the Environment, University of Queensland, St. Lucia, QLD 4072, Australia; zichen.qiao@student.uq.edu.au; 2 Independent Researcher, Yubei District, Chongqing 404100, China; longy6358@gmail.com; 3 Shizhu Tujia Autonomous County Traditional Chinese Medicine Hospital, Shizhu Tujia Autonomous County, Chongqing 409100, China

**Keywords:** *Azemiops feae*, snakebite, neurotoxicity, coagulopathy, antivenom

## Abstract

*Azemiops feae* (Fea’s viper) is a phylogenetically distinctive Asian viper with poorly defined medical significance, and human envenomations remain rarely reported in the English-language literature. We describe a new case of *A. feae* envenoming from Chongqing, China, and present a scoping review of published clinical case reports and case series to better characterize its epidemiology, clinical manifestations, and management. A 53-year-old male developed marked local pain and swelling following a bite to the hand, accompanied by transient neurotoxic symptoms, as well as mild hypofibrinogenemia. Treatment with a single vial of *Gloydius brevicaudus* monovalent antivenom was followed by clinical improvement and full recovery. Review of the literature identified nine previously published studies from China and one captive case from Europe. Envenoming typically occurred during agricultural activities, most commonly affected the lower extremities, and was characterized by prominent local effects with occasional mild neurotoxic features and inconsistent, generally mild coagulation abnormalities. Antivenom use was highly variable, involving multiple heterologous monovalent antivenoms, and outcomes were uniformly favourable regardless of antivenom administration. Collectively, available evidence indicates that *A. feae* envenoming is usually self-limited, with predominantly local effects and infrequent, mild systemic involvement. However, the absence of species-specific antivenom and the heterogeneity of current treatment practices highlight the need for systematic venom characterization and functional antivenom efficacy studies to inform evidence-based clinical management.

## 1. Introduction

Commonly known as Fea’s viper, *Azemiops feae* is a medium-sized, fossorial viperid snake characterized by a secretive and cryptic lifestyle [1,2]. The species inhabits cool, moist montane environments across northern Myanmar, northern Vietnam, and southern China [1,2,3]. In China, its distribution extends from Yunnan in the west to Zhejiang in the east and northward to Shaanxi [1,3]. *Azemiops* represents an enigmatic lineage within the family Viperidae and is currently placed in the monotypic subfamily Azemiopinae, that its basal to the pitviper clade [2,4,5].

**Figure 1:**
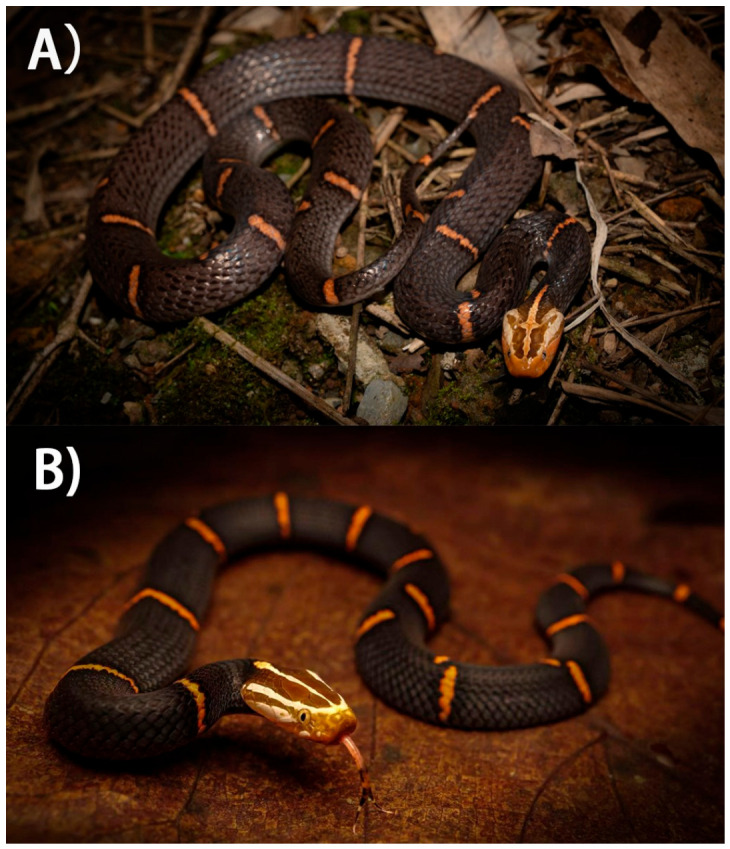
Representative colour morphs of the *Azemiops feae–kharini* complex. (**A**) “White-headed” form photographed in Guangdong Province, China (photo by: Yong Tang). (**B**) “Black-headed” form photographed in Yunnan Province, China (photo by: Deming Shen).

Orlov et al. [2] proposed the recognition of a second species, *A. kharini*, based primarily on head coloration (white-headed versus black-headed forms; Figure 1) and additional morphological and osteological characters; however, this taxonomic treatment did not include genetic analyses. Subsequent molecular studies demonstrated that specimens attributed to *A. kharini* are nested within *A. feae* [6], and intermediate phenotypes combining diagnostic features of both forms have been reported [7]. Accordingly, the Reptile Database currently recognizes *A. feae* as the sole valid species of the genus.

Given the unresolved taxonomic status of *Azemiops* and the inability to verify the identity of individual specimens involved in the present study and previously published studies reviewed, we adopt a conservative taxonomic approach and follow the nomenclature of Uetz et al. [3], referring to all *Azemiops* specimens herein as *A. feae*. We do not attempt to address or resolve the underlying taxonomic controversies in this study.

## 2. Results

### 2.1. Case Report

**Figure 2:**
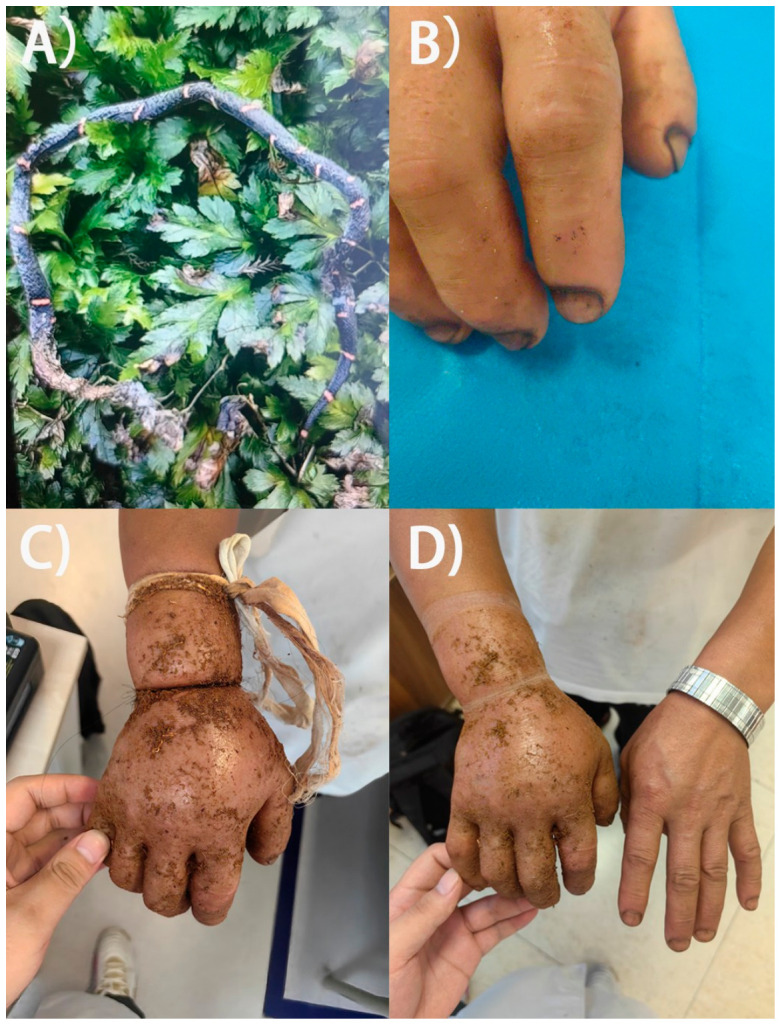
Clinical features of *Azemiops feae* envenoming reported in this study. (**A**) Photograph of the killed offending snake later identified as *A. feae*. (**B**) Fang marks on the right index finger at the bite site. (**C**) Right hand showing constrictive first-aid measures using strands of hair and a cloth band, with marked local swelling. (**D**) Right hand after removal of the hair and cloth band, showing marked local swelling.

On 19 July 2025, at approximately 16:00, a 53-year-old male was bitten on his right index finger while working in his farmland in Shizhu County, Chongqing Municipality, China. The patient killed and photographed the offending snake at the time of the incident, and the image was later reviewed upon presentation to hospital. Based on dorsal coloration and body shape, the snake was identified as a Fea’s viper (*Azemiops feae*) (Figure 2A).

Immediately after the bite, the patient experienced acute pain and progressive swelling of the right hand. As first-aid, he applied multiple tight ligatures: a strand of hair at the base of the right index finger, another strand of hair at the right wrist, and a cloth band approximately 10 cm proximal to the wrist (Figure 2C). He also topically applied an herbal poultice (unknown ingredients) mixed with rice wine to the swollen area. Pain and swelling continued to worsen overnight, prompting removal of the ligature at the finger due to severe pain. With no improvement by the following afternoon, the patient sought medical care and arrived at Shizhu Tujia Autonomous County Traditional Chinese Medicine Hospital on July 20 (Day 0), at 16:48 (approximately 24.8 h post-bite).

Upon arrival, the patient was alert and afebrile. Vital signs were stable: temperature 36.7 °C, pulse 80 beats/min, respiratory rate 20 breaths/min, and blood pressure 130/98 mmHg. Examination revealed fang marks on the dorsal surface of the right index finger (Figure 2B), marked swelling of the hand and wrist, restricted finger movement, and significant tenderness. No lymphadenopathy was detected in the right axilla. The patient denied nausea, vomiting, chest tightness, dyspnea, dizziness, headache, abdominal pain, or malaise.

Initial management included removal of all constrictive bands (Figure 2D), wound cleansing, oxygen supplementation, ECG monitoring, and intravenous administration of dexamethasone (10 mg), mannitol (125 mL), vitamin C (2 g) and pantoprazole (40 mg). Tetanus prophylaxis was also administered intramuscularly. Traditional Chinese medicine (TCM) treatments were applied concurrently: small skin incisions between the right index and middle finger, approximately at the depression proximal to the second dorsal interosseous muscle for decompression and external application of a mixture of herbal powders (including *Lobelia chinensis*, *Scutellaria barbata*, *Paris polyphylla*, and others). It must be noted that dexamethasone, mannitol, vitamin C, pantoprazole, and traditional Chinese medicine (TCM) treatments all lack evidence of effectiveness for the treatment of snakebite.

Initial laboratory testing performed at 17:46, approximately 25.5 h post bite, showed normal platelet count and predominantly normal coagulation profile (Table 1), except for mildly reduced fibrinogen (1.92 g/L; reference: 2.0–4.0 g/L). D-dimer was within normal limits (0.29 µg/mL; reference: 0–0.5 µg/mL). At 20:00, 27 h post envenomation, one vial of *Gloydius brevicaudus* monovalent antivenom (GbMAV; 6000 U/10 mL) was administered. At 20:30, the patient developed neurotoxicity symptoms including palpitations, perioral numbness, drowsiness, and bilateral ptosis. Vital signs and oxygen saturation remained stable, and no rash or airway compromise developed. The patient was observed closely with supplemental low-flow oxygen.

On the second morning (21 July, Day 1), the patient reported marked improvement in swelling and discomfort. Palpitations, numbness, drowsiness, and ptosis had resolved. In the absence of progressive neurotoxic manifestations, no additional antivenom was administered. Supportive therapy continued with intravenous administration of dexamethasone, mannitol, vitamin C, and pantoprazole at the same dosages used on Day 0. TCM therapy, including internal herbal decoctions aimed for “heat-clearing and detoxicating”, was continued as per local practice.

By the third morning (22 July, Day 2), swelling had further improved, and no recurrence of palpitations, numbness, drowsiness, or ptosis was reported. No further antivenom was administered. The dexamethasone dose was reduced to 5 mg, and mannitol and vitamin C were continued in dosages consistent with previous days, and mannitol was discontinued. Repeat laboratory testing at 08:00, approximately 64 h post envenomation revealed mildly elevated white blood cell count (14.27 × 10^9^/L), mildly reduced red blood cell count (4.29 × 10^12^/L), and stable platelets. Coagulation testing showed slight prolongation of thrombin time (21.9 s) and decreased fibrinogen (1.39 g/L), consistent with mild hypofibrinogenemic coagulopathy (Table 1).

On the fourth day (23 July, Day 3), swelling had completely resolved and the skin incision sites had healed. The patient requested discharge, as clinical signs had resolved and laboratory abnormalities on previous day were mild, he was discharged with instructions for rest and follow-up observations on potential recurrence of symptoms.

### 2.2. Case Report Literature Review

**Figure 3:**
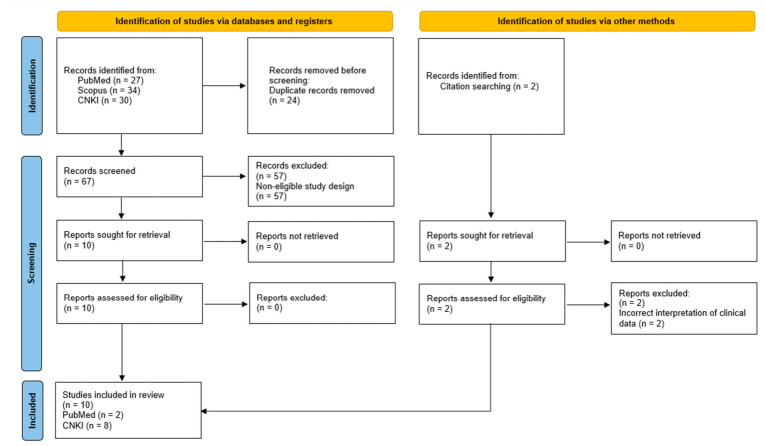
PRISMA flow diagram summarizing the literature search and study selection process for this review. Template adapted from the PRISMA 2020 Flow Diagram (https://www.prisma-statement.org/prisma-2020-flow-diagram). Accessed on 1 March 2026.

The keyword searches retrieved a total of 91 records, of which 24 were identified as duplicates and removed. Screening of titles and abstracts resulted in the exclusion of a further 57 records that did not report clinical case reports or case series of *A. feae* envenoming. Citation searching identified two additional publications; however, these were duplicate references that briefly mentioned the identical three cases of *A. feae* bites without providing any case descriptions and were therefore excluded. Consequently, 10 studies, comprising two publications in English and eight in Chinese, met the inclusion criteria and were included in the review. The study identification, screening, and inclusion process is summarized in a PRISMA flow diagram (Figure 3).

**Figure 4:**
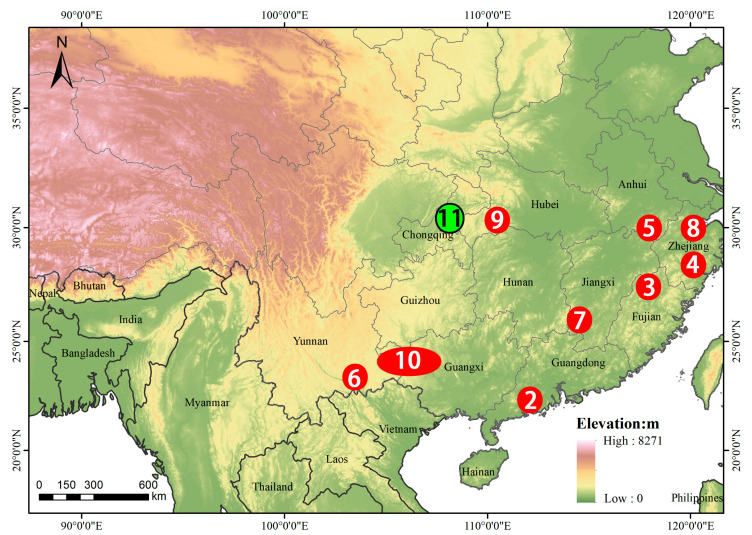
Topographic map showing reported localities of human envenomation by wild *Azemiops feae–kharini* complex (captive snakebite in Valenta, Stach, Stourac, Kadanka and Michalek [8] not included). Elevation is represented using a colour gradient (0–8271 m), with national, provincial and municipal boundaries labelled. Red circles denote localities of single envenomation cases reported in the literature; the green circle indicates the locality of the single case reported in this study. The dashed circle outlines the study area of the case series reported by Yang [17]. Numbers in each point and the oval circle correspond to the studies summarized in Table 2.

**Figure 5:**
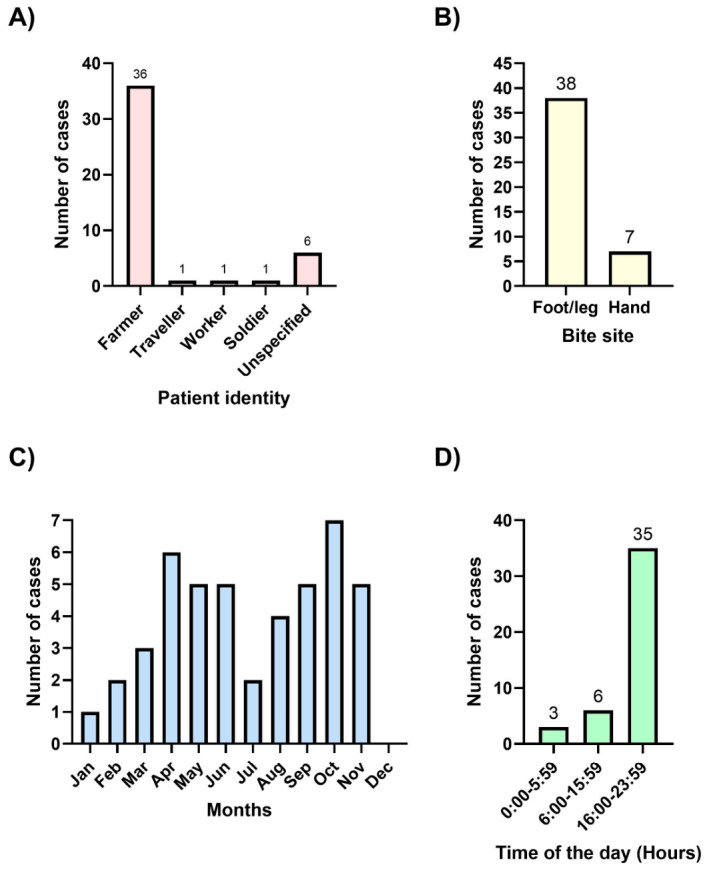
Epidemiological patterns of *Azemiops feae* envenoming based on cases reported in the literature and a single case from the present study. The captive bite case described by Valenta et al. [8] is excluded. (**A**) Social identity of patients. (**B**) Distribution of bite sites between hand and foot/leg. (**C**) Monthly distribution of incidents. (**D**) Time-of-day distribution of incidents. The case presented in Guo et al. [9] is excluded in (**D**) as time of the day of the incident is missing.

Of the 10 studies included, 9 were single case reports, and 1 was a case series comprising 36 cases of *A. feae* envenoming. The full text of each report was reviewed, and key epidemiological and clinical information from all published cases (Cases 1–10), together with the single case reported in the present study (Case 11), are summarized in Table 2. The geographic localities of all studies reporting bites by wild snakes are shown in Figure 4, and aggregated epidemiological patterns are presented in Figure 5. The case reported by us is the green dot on Figure 4; GPS 29.998120, 108.113830.

Cases of *A. feae* envenoming reported in the literature, together with the single case from the present study, were distributed broadly across southern China (Figure 4) and exhibited consistent epidemiological patterns with respect to patient identity, bite site, seasonality, and time of day (Figure 5). Most patients were identified as farmers (36 cases), while only isolated cases involved travellers, workers, or soldiers; patient occupation was unspecified in six cases (Figure 5A). Bites predominantly affected the lower extremities, with 38 cases involving the foot or leg, compared with seven cases involving the hand (Figure 5B). Incidents were recorded throughout the year, with cases reported in all months except December; higher numbers were observed between April and June and again between September and November (Figure 5C). Envenoming occurred across all time periods of the day, with the highest frequency recorded between 16:00 and 23:59 (35 cases), followed by 06:00–15:59 (six cases), and 00:00–05:59 (three cases) (Figure 5D).

Based on the data summarized in Table 2, the clinical presentation of *A. feae* envenoming was generally characterized by prominent local effects with variable systemic symptoms. Local pain and swelling at the bite site were the most consistent features, reported in nearly all cases across single case reports and the larger case series by Yang [17]. In several cases, local manifestations were mild, with no signs of systemic envenoming [11,13,15,16,17]. Severe tissue damages or necrosis were not reported in any studies.

Systemic manifestations were comparatively uncommon but diverse when present. Signs of suspected neurotoxic envenoming, including dizziness [10,12,17], limb numbness [10], blurred vision [8,12,14], muscle fasciculations [8], and ptosis [12,14,17], were reported sporadically across cases. Other symptoms such as nausea [12,14] and chills [10], were also occasionally documented. Notably, no cases progressed to respiratory failure.

Abnormalities in coagulation parameters were inconsistently reported in earlier case reports; however, among studies that assessed coagulation parameters, mild hypofibrinogenemia (none with fibrinogen dropped below the minimum detection limit) emerged as the most frequently observed abnormality in Guo et al. [9] and Yang [17]. In the largest case series reported by Yang [17], reduced fibrinogen levels were documented in 16 of 36 patients, while other coagulation parameters largely remaining within normal ranges. Renal involvement was rare, with only a single elderly patient exhibiting elevated creatinine levels [14].

Antivenom use varied substantially between cases and institutions. Some patients did not receive antivenom and nonetheless recovered uneventfully [8,10,13,17], whereas others were treated with one or more vials of heterologous monovalent antivenoms. All four commercially available monovalent antivenoms in mainland China had been used to treat *A. feae* envenoming, including DaMAV *Deinagkistrodon acutus* pitviper snake venom monovalent used in Fu et al. [14], Guo et al. [9], and Wang and Cheng [12]; GbMAV *Gloydius brevicauda* pitviper snake venom monovalent in Guo et al. [9], Wang and Cheng [12], Xiong et al. [15], and Yu et al. [16]; BmMAV *Bungarus multicinctus* elapid snake venom monovalent in Fu et al. [14], Guo et al. [9], Xiong et al. [15], Yang [17], and Yu et al. [16]; and NaMAV *Naja atra* elapid snake venom monovalent in Chen [11], either alone or in combination. The antivenom administrations were primarily based on local progression, systemic symptoms, or laboratory findings and the types of antivenom available, rather than any standardized criteria. Despite this heterogeneity in management, outcomes were uniformly favourable: all patients recovered, hospitalization durations were generally short (≤8 days in the largest case series by Yang [17]), and no fatalities or permanent sequelae were reported.

## 3. Discussion

This study expands the limited clinical literature on *Azemiops feae* envenoming by integrating a new case with previously published reports, allowing a clearer assessment of the epidemiological and clinical characteristics of this poorly known species. Across the available cases, envenoming by wild *A. feae* was confined to southern China and occurred predominantly in montane regions, consistent with the known distribution and ecological preferences of the species [1,3]. Envenoming events were strongly associated with outdoor occupational activities, particularly farming, reflecting accidental encounters during manual work and mirroring global trends in snakebite epidemiology [18]. Most bites involved the distal lower limbs, consistent with general snakebite patterns reported in China and globally [19,20,21,22]. However, as some studies have documented a higher frequency of bites to the upper limbs [23,24,25], further documentation of envenomation by *A. feae* is needed to better characterize bite patterns in this poorly known species. Cases occurred throughout the year, with peaks between April and June and between September and November, lower frequency between July and August may be explained by *A. feae*’s preference for cool and moist conditions and aligns with natural history observations from wild and captive specimens [1]. Envenoming was documented throughout the day, with a distinct nocturnal peak, suggesting that bites most frequently result from nocturnally active individuals than disturbance of resting or concealed snakes. Collectively, these findings indicate that *A. feae* envenoming represents an occupational and environmentally mediated risk, disproportionately affecting rural populations engaged in agricultural activities within suitable montane habitats during the evening hours.

The clinical features of *A. feae* envenoming align only partially with the venom composition and activities reported from experimental venom studies. The predominance of local pain and swelling across nearly all reported cases is consistent with the high abundance of phospholipase A_2_ (PLA_2_) and presence of other toxins known to induce pain and swelling [26,27], such as snake venom serine proteases (SVSPs) and snake venom metalloproteinases (SVMPs) in *A. feae* venom [28,29,30,31]. Conversely, the absence of severe tissue necrosis or extensive local tissue destruction accords with experimental findings demonstrating low or absent cytotoxic, myotoxic, and proteolytic tissue-damaging activities in *A. feae* venom [28,32].

Neurotoxic effects of *A. feae* venom are likely mediated primarily by azemiopsin, a polypeptide shown to block nicotinic acetylcholine receptors [33]. These alpha-neurotoxic peptides *de novo* evolved from within the propeptide region of the C-type natriuretic peptide [34]. Genetic analyses suggest that these peptides share a molecular ancestor with the waglerin peptides from *Tropidolaemus* species, which are also alpha-neurotoxic [35,36,37,38,39]. The reports of neurotoxic manifestations following envenomations by *A. feae*, including ptosis, blurred vision, limb numbness, and muscle fasciculations, corresponds with in vivo lethality studies in mice and rabbits [28,32,33], and in vitro evidence demonstrating potent inhibition of nicotinic acetylcholine receptors across multiple vertebrate taxa, including humans [40]. However, the generally mild nature of neurological symptoms, together with the absence of respiratory failure in all reported cases, suggests that although neurotoxic components are biologically active, their clinical relevance in human envenoming is typically limited. This is consistent with data showing that, consistent with dietary preferences, *A. feae* neurotoxicity is highly selective amphibian nicotinic acetylcholine receptors than mammals, being 1.9 times more potent on amphibian receptors than rodents, and 3.44 times more potent on amphibian receptors than on humans [40].

In contrast, the occasional reports of mild hypofibrinogenemia observed in clinical cases are not readily explained by existing venom studies. Experimental investigations have consistently failed to demonstrate coagulotoxic activity of *A. feae* venom, both in vitro using human plasma and fibrinogen [41], and in vivo in mice and rabbit models [28,32]. While interspecific differences in coagulotoxic venom activity may partly explain discrepancies between animal models and human clinical findings [42], the absence of detectable effects on human plasma and fibrinogen in vitro suggests alternative explanations. One possibility is intraspecific venom variation, particularly in the relative abundance of SVMPs and SVSPs, two toxin families known to act on fibrinogen through non-clotting cleavage or pseudo-procoagulant (thrombin-like) manners [43,44]. Such variation has been documented in *Azemiops* venoms, with a northern Vietnamese specimen exhibiting a significantly higher abundance of SVSPs compared with Chinese specimens from Sichuan and Zhejiang [28,31]. Consistent with this is the low levels of activity on fibrinogen for the Yunnan sample used in Debono et al. [41]. Importantly, fibrinogen depletion in reported Chinese cases was consistently mild, with no values falling below assay detection limits and no progression to more clinically significant complete coagulopathy. Collectively, these observations indicate that coagulation disturbance is neither a dominant nor a consistent feature of *A. feae* envenoming. Nevertheless, given its recurrent documentation across multiple studies, routine monitoring of coagulation parameters remains a prudent component of clinical management.

Despite all four commercially available monovalent antivenoms having been used in reported cases of *A. feae* envenoming [11,14,17], the existing clinical data do not allow reliable assessment of antivenom effectiveness or potency against this species. Some patients recovered uneventfully without receiving antivenom [8,10,13,17], whereas others improved following administration of one or multiple vials of non-species-specific monovalent antivenoms. This heterogeneity, together with the generally mild clinical course observed in many cases, makes it impossible to distinguish spontaneous recovery from treatment-related effects based solely on existing case reports and case series.

Experimental data provide limited but potentially informative insights. Using in vitro ELISA assays, Zheng et al. [28] demonstrated that all four Chinese monovalent antivenoms exhibit immunorecognition of *A. feae* venom, with *Gloydius brevicaudus* monovalent pitviper snake antivenom (GbMAV) showing the strongest binding capacity. On this basis, the authors recommended GbMAV as a potential treatment option for *A. feae* envenoming [28]. Clinically, *Bungarus multicinctus* monovalent elapid snake antivenom (BmMAV) has been administered in patients with neurotoxic features following *A. feae* bite [14,17], while *Deinagkistrodon acutus* monovalent pitviper snake antivenom (DaMAV) has been recommended in Chinese national guidelines for the management of “haemotoxic” envenoming [19]. However, immunorecognition and empiric clinical use do not equate to proven neutralization or clinical effectiveness. Overall, the available evidence remains insufficient to establish the optimal antivenom choice or dosing strategy for *A. feae* envenoming. Well-designed future studies integrating functional neutralization assays and systematically documented clinical outcomes are needed to evaluate the efficacy and effectiveness of both Chinese and global antivenoms against *A. feae* venom. As none of the snakes used in the immunizing mixture of the above antivenoms are close relatives of *A. feae* and there was a similarity of outcomes, this suggests that the antivenoms had minimal, if any, cross-reactivity with the *A. feae* venom, and that the consistent recoveries were not linked to antivenom usage.

## 4. Conclusions

In conclusion, this study expands the limited clinical knowledge of *A. feae* envenoming by integrating a newly documented case with a comprehensive synthesis of previously published reports, many of which are in Chinese and therefore less accessible to the international research community. The new case adds valuable clinical detail to a sparsely documented and understudied species and reinforces that *A. feae* envenoming is typically characterized by prominent local effects with occasional, generally mild systemic manifestations. By collating and analyzing both English- and Chinese-language sources, this study helps bridge an important language barrier in snakebite research, providing a more complete and globally accessible overview of the epidemiology, clinical features, and treatment practices associated with *A. feae* envenoming. These findings highlight persistent knowledge gaps in venom activities of *A. feae* and antivenom efficacy against *A. feae* envenoming, underscoring the need for future studies combining standardized clinical case reports with experimental venom and antivenom research.

## 5. Materials and Methods

### 5.1. Case Report

We report a case of confirmed *Azemiops feae* envenomation based on medical records of Shizhu Tujia Autonomous County Traditional Chinese Medicine Hospital, Shizhu County, Chongqing Municipality, China.

### 5.2. Case Report Literature Review

We searched the PubMed and Scopus databases on 1 December 2025 using the keywords “*Azemiops*” and “Fea’s viper,” and the China National Knowledge Infrastructure (CNKI) using as keywords the Chinese common names of *Azemiops* “白头蝰” and “黑头蝰”. Titles and abstracts of all retrieved records were screened, and only clinical case reports or case series describing envenoming by confirmed *Azemiops feae* were included. In addition, the reference lists of all screened articles were manually reviewed to identify further potentially relevant publications. Toxicological studies, including venom composition analyses and in vitro or in vivo venom activity experiments, were excluded from this mini review. The study selection process and numbers of included and excluded records were reported in accordance with the PRISMA-Scoping Review guidelines.

## Figures and Tables

**Table 1:** Parameters from coagulation tests performed post *Azemiops feae* envenomation in Shizhu, Chongqing, China. PT = prothrombin time; TT = thrombin time; APTT = activated partial thromboplastin time; INR = international normalized ratio; FIB = fibrinogen; D-dimer = fibrin degradation product. GbMAV = *Gloydius brevicaudus* monovalent antivenom. ↑ = above reference range; ↓ = below reference range.

Test	25.5 h	27 h	64 h	Reference Range
PT (s)	11.4	GbMAV (1 vial)	12.5	10–14
TT (s)	19.6		21.9 ↑	14–21
APTT (s)	26.5		25.5	22–38
INR	0.95		1.04	0.8–1.25
FIB (g/L)	1.92 ↓		1.39 ↓	2–4
D-dimer (mg/L)	0.29		0.46	0–0.5

**Table 2:** Reported clinical manifestations of *Azemiops feae-kharini* complex envenoming based on this study and published clinical case reports and case series. Abbreviations: DaMAV = *Deinagkistrodon acutus* monovalent antivenom; GbMAV = *Gloydius brevicaudus* monovalent antivenom; BmMAV = *Bungarus multicinctus* monovalent antivenom; NaMAV = *Naja atra* monovalent antivenom. The date of envenoming is designated as Day 0. Case from this study is shown with green highlight.

No.	Location	No. of Cases	Patient Profile	Bite Site	Clinical Manifestations	Antivenom	Outcome	Remarks	Reference
1	Czech Republic (captive)	1	43-year-old male	Left forefinger	Local pain and swelling; muscle fasciculations; blurred vision	None	Full recovery; discharged Day 14	Captive snakebite	Valenta et al. [8]
2	Guangdong, China	1	34-year-old female	Right ankle	Pain, swelling; finger/toe stiffness; mild hypofibrinogenemia	2 vials DaMAV; 2 vials BmMA; 2 vials GbMAV	Recovered; discharged Day 5	Patient allergic to GbMAV	Guo et al. [9]
3	Fujian, China	1	35-year-old male	Right calf	Pain, swelling; dizziness; limb numbness; chills	None	Recovered; discharged Day 4	Coagulation not tested; possibly allergic to venom	Tang et al. [10]
4	Zhejiang, China	1	47-year-old female	Right ankle	Local pain and swelling	3 vials NaMAV	Recovered; discharged Day 8	Coagulation not tested	Chen [11]
5	Anhui, China	1	49-year-old male	Right ankle	Pain, swelling; nausea; dizziness; blurred vision; ptosis	2 vials DaMAV; 1 vial GbMAV	Recovered; discharged Day 5	Coagulation not tested	Wang and Cheng [12]
6	Yunnan, China	1	27-year-old male	Right heel	Mild local swelling only	None	Recovered; discharged Day 7	Coagulation not tested, wound incision performed	Hou et al. [13]
7	Jiangxi, China	1	77-year-old male	Left ankle	Pain, swelling; nausea; blurred vision; ptosis; elevated creatinine	2 vials DaMAV; 2 vialsBmMAV	Improved; discharged Day 21	Coagulation not tested, renal involvement suspected	Fu et al. [14]
8	Zhejiang, China	1	65-year-old female	Right foot dorsum	Local swelling	1 vial GbMAV; 1 vial BmMAV	Recovered; discharged Day 1	Wound incision performed	Xiong et al. [15]
9	Hubei, China	1	54-year-old male	Left foot	Pain, swelling	2 vials GbMAV; 2 vials BmMAV	Recovered; discharged Day 1	Coagulation not tested	Yu et al. [16]
10	Yunnan and Guangxi, China	36	21 males, 15 females; mean age 41 years	Foot (30 patients), hand (6)	Pain (35), swelling (all); mild hypofibrinogenemia (16); dizziness (2); ptosis (1)	21 patients treated with antivenom (unspecified type and amount); BmMAV (unspecified amount) for the ptosis case	All recovered within ≤8 days	Coagulation parameters tested for all patients	Yang [17]
11	Chongqing, China	1	53-year-old male	Right index finger	Pain, swelling; palpitation; numbness around the mouth; drowsiness; ptosis; mild hypofibrinogenemia	2 vials GbMAV	Recovered, discharged Day 5	Presented to hospital one day after bite	This study

## Data Availability

The original contributions presented in this study are included in the article. Further inquiries can be directed to the corresponding authors.
